# Bone marrow CCR3 dictates eosinophil lineage commitment of CD34⁺ progenitors to orchestrate allergic rhinitis: A composite study

**DOI:** 10.1371/journal.pone.0351726

**Published:** 2026-06-22

**Authors:** Zhi-qiang Zhang, Meng-yi Wei, Jia-le Bei, Jing-yang Li, Mei-na Dai, Yin-li Jiang, Ya-ting Xiao, Ying Zhang, Xinhua Zhu

**Affiliations:** 1 Department of Otorhinolaryngology, Head and Neck Surgery, The Second Affiliated Hospital, Jiangxi Medical College, Nanchang University, Nanchang, China; 2 The First School of Clinical Medicine, Southern Medical University, Guangzhou, Guangdong, China; 3 Department of Clinical Medicine, The First Clinical Medical College, Nanchang University, Nanchang, China; Centre de Recherche en Cancerologie de Lyon, FRANCE

## Abstract

**Background:**

Allergic rhinitis (AR) is a common Th2-mediated inflammatory disease of the nasal mucosa, in which eosinophils serve as pivotal effector cells. The CCR3 receptor, specific for eotaxin, plays a critical role in allergic inflammation. However, the role of CCR3 in the differentiation of bone marrow CD34 ⁺ progenitor cells into eosinophils and its contribution to AR pathogenesis remains incompletely defined.

**Objective:**

This study aimed to investigate whether bone marrow cell-specific CCR3 deletion is associated with altered CD34 ⁺ progenitor abundance, eosinophil-lineage–related responses, and allergic inflammation in a murine model of allergic rhinitis.

**Methods:**

An integrative approach was employed. Bioinformatic analyses of transcriptomic data from CCR3-deficient mice, including differential expression screening, WGCNA, functional enrichment, and six machine learning algorithms, were used to identify candidate genes associated with CCR3 deletion. A bone marrow cell-specific CCR3 conditional knockout (CCR3-CKO) mouse model was generated and subjected to an ovalbumin-induced AR protocol. Nasal symptoms, body weight, nasal mucosal histopathology (H&E, PAS), serum cytokine/mediator levels (IL-5, eotaxin, ECP, EPO), and immune cell populations were assessed. Flow cytometry quantified CD34 ⁺ progenitors, CD34 ⁺ CCR3 ⁺ progenitors, and eosinophils in bone marrow, peripheral blood, and nasal lavage fluid. In vitro transwell migration and eosinophil colony-forming assays were performed to evaluate progenitor cell function.

**Results:**

Bioinformatic and machine-learning analyses identified CD34 as a candidate hub gene associated with CCR3 deletion. In the AR model, CCR3-CKO mice showed reduced nasal symptom scores, less inflammatory-cell infiltration, and attenuated tissue injury, with a trend toward reduced allergy-associated weight loss. CCR3 deletion was associated with lower CD34 mRNA and protein levels in bone marrow and peripheral blood, as well as reduced proportions of CD34 ⁺ progenitors, CD34 ⁺ CCR3 ⁺ progenitors, and eosinophils across the analyzed compartments. Serum IL-5, ECP, and EPO levels were decreased, whereas eotaxin levels were increased. In vitro, CCR3 deficiency showed a modest reduction in the migratory response of CD34 ⁺ progenitors to eotaxin and partially reduced IL-5/eotaxin-associated eosinophil colony formation.

**Conclusion:**

Bone marrow cell-specific CCR3 deletion was associated with reduced eosinophil-lineage–related progenitor abundance, decreased eosinophilic inflammation, and partial improvement of AR-associated phenotypes. These findings suggest that the eotaxin/CCR3 axis may contribute to hematopoietic and eosinophil-lineage regulation in allergic inflammation. However, the direct cell-intrinsic regulation of CD34 expression and the relative contribution of IL-5-dependent pathways require further investigation.

## 1. Introduction

Allergic rhinitis (AR), commonly affecting atopic individuals following allergen exposure, involves non-infectious inflammation of the nasal mucosa. This condition arises mainly from IgE-mediated release of inflammatory mediators and engages a range of immune cells and cytokines [1]. Typical clinical symptoms include sneezing, nasal obstruction, watery nasal discharge, and itching [2]. From an immunological perspective, AR reflects a localized Th2-type immune response in the nasal mucosa that depends on IgE [3,4]. Within this process, eosinophils (EOS) perform essential roles as effector cells [5].

Derived from multipotent CD34^+^ progenitor cells [6], eosinophils belong to the granulocyte lineage and mainly reside in mucosal tissues of the respiratory, digestive, and urogenital systems. Their morphology is distinctive, marked by a bilobed nucleus and numerous characteristic granules. When exposed to antigen, CD34^+^ progenitor cells located in the bone marrow undergo proliferation and maturation into eosinophils. These cells subsequently enter the bloodstream and travel to the airways. At the same time, a proportion of CD34^+^ progenitors leave the bone marrow and use the circulation to reach various peripheral tissues [7,8]. Apart from functioning as terminal effector cells, eosinophils also operate as specialized antigen-presenting cells and take part in immune regulation and tissue remodeling. As a result, they remain a central subject in AR investigations [9].

A key member of the CC chemokine family, eosinophil chemokine mediates the rapid movement of eosinophil progenitor cells out of the bone marrow and into the blood [10]. This chemokine then guides these cells toward regions of allergic inflammation, where they substantially influence the ensuing inflammatory events. It is worth emphasizing that CCR3 functions as the exclusive and highly specific receptor for this chemokine [11]. Our earlier investigations, reinforced by substantial experimental evidence, have established CCR3 as an important immune-related marker gene involved in allergic processes [5,12–14]. Studies in asthma and atopic dermatitis indicate that the eosinophil/CCR3 axis supports eosinophil recruitment to cutaneous and pulmonary tissues [15,16]. Thus, interfering with the eotaxin/CCR3 pathway could represent a valuable new treatment approach for allergic conditions.

Recent studies have highlighted the important role of the eotaxin/CCR3 axis in allergic inflammation, particularly in regulating eosinophil recruitment and activation. In our previous work using a BALB/c CCR3-deficient mouse model, we demonstrated that CCR3 knockout significantly inhibited eosinophil proliferation, migration, and degranulation, thereby alleviating inflammatory responses in allergic rhinitis. That study also showed that CCR3 deficiency was associated with a reduction in CD34 + progenitor cells in bone marrow and peripheral blood, suggesting that CCR3 signaling may influence upstream hematopoietic processes involved in eosinophil development. However, the mechanisms linking CCR3 signaling to hematopoietic progenitor cell dynamics remain poorly understood. In particular, it is unclear whether CCR3 contributes to allergic inflammation not only by regulating mature eosinophil function but also by influencing the abundance or eosinophil-lineage commitment of CD34 + progenitor cells.

To address this question, the present study employed a bone marrow–specific CCR3 conditional knockout mouse model generated on a C57BL/6 background. By integrating transcriptomic bioinformatics analysis, multiple machine learning algorithms, and experimental validation in vivo and in vitro, we aimed to identify candidate genes associated with CCR3 deletion and to evaluate whether CCR3 deficiency alters CD34 + progenitor abundance and eosinophil-lineage development during allergic rhinitis. Collectively, this study seeks to provide new insight into the potential role of CCR3 in regulating hematopoietic progenitor dynamics in allergic inflammation and to expand our understanding of the mechanisms linking chemokine signaling with eosinophil-lineage commitment in allergic rhinitis.

## 2. Materials and methods

### 2.1. Data acquisition

Transcriptomic data were obtained from RNA sequencing of allergic rhinitis (AR) mouse models (n = 6 per group) in CCR3(-) VS CCR3(+) gene states.

### 2.2. Identification of CCR3(-)-associated differentially expressed genes (DEGs)

Differential gene expression between control and experimental groups was analyzed using the R package ‘limma’ [17]. Genes with a log2 fold change (log2FC) > 0.5 and p-value < 0.05 were classified as upregulated, while those with a log2FC < −0.5 and p-value < 0.05 were considered downregulated. Results were visualized using volcano plots.

### 2.3. Construction of WGCNA Co-expression networks

Gene co-expression networks were constructed using the WGCNA package in R. Genes with the smallest 50% mean absolute deviation (MAD) were filtered out, and the goodSamplesGenes method was applied to remove outliers. A scale-free co-expression network was built with a soft thresholding power of 10. Modules were identified, and those with a distance less than 0.25 were merged. Genes not assigned to any module were grouped into the grey module.

### 2.4. Functional enrichment analysis

Gene Ontology (GO) enrichment analysis for DEGs was conducted using the clusterProfiler package, covering biological process (BP), cellular component (CC), and molecular function (MF) categories [18]. The top three significantly enriched terms (p < 0.05) from each category were visualized using ggplot2. Kyoto Encyclopedia of Genes and Genomes (KEGG) pathway enrichment was similarly performed and visualized [19–21].

### 2.5. Gene set enrichment analysis (GSEA)

GSEA was implemented via the clusterProfiler package to identify enriched gene sets from MSigDB. Gene sets with a nominal p-value < 0.05 and a q-value < 0.25 were considered significantly enriched and were ranked by their enrichment score (ES).

### 2.6. Protein-protein interaction (PPI) network analysis

The STRING database was used to predict protein-protein interactions, and the resulting network was visualized and analyzed in Cytoscape [22]. Highly interconnected clusters were identified using the MCODE algorithm [23].

### 2.7. Machine learning for feature selection

Six machine learning algorithms were employed to identify candidate genes associated with CCR3 deletion: LASSO & Ridge: Penalized regression methods for variable selection and prediction accuracy [24]; Boruta: A wrapper algorithm for identifying all-relevant features [25]; Random Forest (RF): Used to rank feature importance based on node impurity [26,27]; Support Vector Machine (SVM): A kernel-based method for pattern recognition [28]; XGBoost: A gradient-boosting framework utilizing decision trees [29].

Genes commonly identified across these methods were considered candidate genes associated with CCR3 deletion.

### 2.8. Hub gene validation and diagnostic value assessment

Hub gene expression was compared between CCR3 knockout and control groups using T-tests, with results visualized via ggplot2. The diagnostic potential of these genes was evaluated by receiver operating characteristic (ROC) curve analysis using the pROC package. An area under the curve (AUC) > 0.7 was considered indicative of diagnostic value [30]. However, because CIBERSORT was originally developed for human immune signatures, this analysis was used as an exploratory deconvolution approach for murine transcriptomic data and interpreted with caution.

### 2.9. Immune infiltration analysis

Immune cell proportions were estimated from gene expression data using the CIBERSORT algorithm [31]. Stacked bar plots, correlation heatmaps, and boxplots were generated to compare immune cell profiles between CCR3(-) and control groups. Spearman’s correlation was used to assess relationships between immune cell subtypes.

### 2.10. Experimental animals

Male C57BL/6 bone marrow cells with conditional knockout of the CCR3 gene (CCR3-CKO) and control wild-type (WT) mice (litter-matched wild-type mice) used in the experiment were aged 6–8 weeks. During the study period, mice were housed at the SPF transgenic animal centre of the Institute of Translational Medicine, Jilin University. All experimental procedures were conducted in accordance with the approval of the Welfare and Ethics Committee of the Institute of Translational Medicine, Jilin University.

### 2.11. Generation of conditional knockout (CCR3-CKO) mice in bone marrow cells

The CCR3-RV vector was constructed and introduced into CCR3-BAC-loxP host bacteria. The RV-CCR3-loxP plasmid was modified to remove the Neo marker, yielding a CCR3 targeting vector. This targeting vector was electroporated into ES cells, and ES clones exhibiting correct homologous recombination were selected. Following blastocyst injection, heritable flox-homozygous mice with conditional CCR3 gene knockout were obtained. These heterozygous mice were then crossed with bone marrow cell-specific Cre tool mice to generate a model with conditional knockout of the CCR3 gene in bone marrow cells. The detailed procedure is outlined in S1 Fig.

### 2.12. Genotyping of mice

Genotyping for CCR3 was described in a previous study [32]. Briefly, DNA was extracted from offspring mice, amplified via specific PCR, and genotyping determined by agarose gel electrophoresis of PCR products.

### 2.13. Allergic rhinitis model and symptom assessment

Mice were divided into four groups (n = 6 per group): CCR3-CKO AR (CKO-OVA), CCR3-CKO Blank (CKO-Control), WT Blank (WT-Control), WT AR (WT-OVA). The AR model was established by intraperitoneal sensitization with ovalbumin (OVA) on days 0, 7, and 14, followed by intranasal OVA challenge on days 21–27. Control groups received saline. Nasal symptoms (sneezing and scratching) were recorded for 10 minutes after the final challenge, as previously described [33]. Nasal symptoms were scored according to a previously published standardized scoring protocol, including sneezing and nose-scratching events within 10 min after the final nasal challenge. To minimize subjective bias, all behavioral observations and symptom scoring were performed by independent investigators who were blinded to mouse group allocation. However, a formal inter-observer variability analysis was not performed.

### 2.14. Measurement of mouse body weight

The body weight of mice was recorded before each intraperitoneal sensitisation and nasal provocation.

### 2.15. Sample collection

After anesthesia, blood was collected from the jugular vein. Nasal lavage fluid (NALF), nasal mucosa, and bone marrow were harvested. Bone marrow mononuclear cell suspensions were prepared by flushing femurs with cold PBS and filtering through a 200-μm strainer. Peripheral blood leukocytes were isolated using red blood cell lysis buffer.

### 2.16. Western blotting

Proteins were extracted from bone marrow cells, peripheral blood leukocytes, and nasal mucosa using RIPA lysis buffer (TransGen, Beijing, China) supplemented with PMSF and protease inhibitor cocktail (100:1:1). Samples were homogenized using a tissue grinder, incubated on ice, and centrifuged at 14,000 × g for 10 min at 4°C. The supernatants were collected, and protein concentrations were determined using the BCA protein assay. Equal amounts of protein were mixed with 6 × loading buffer, denatured at 100°C for 8 min, separated on 10% SDS-PAGE gels with a 5% stacking gel, and transferred onto NC membranes at 100 V for 80 min on ice. Membranes were blocked with 5% non-fat milk for 2 h at room temperature and incubated overnight at 4°C with primary antibodies against CCR3, CD34, and GAPDH (Affinity, Jiangsu, China). After washing three times with TBST (10 min each), membranes were incubated with the corresponding HRP-conjugated secondary antibodies for 120 min at room temperature. Protein bands were visualized using ECL reagent and imaged using a chemiluminescence imaging system. Band intensities were quantified and normalized to GAPDH.

### 2.17. qPCR analysis of CD34 mRNA

Total RNA was extracted from bone marrow, peripheral blood, and nasal mucosa using Trizol reagent. cDNA was synthesized using a PrimeScript RT reagent kit. qPCR was performed using SYBR Green Master Mix on a QuantStudio system. Primer sequences are listed in Table 1. Reaction components and cycling conditions are detailed in S1.

**Table 1:** Primer Sequences.

Primer Name	Primer Sequence 5’-3’
CD34 Forword primer	ATCCCCATCAGTTCCTACCAAT
CD34 Reverse primer	TGGTGTGGTCTTACTGCTGTC
GAPDH Forword primer	CGCCTGGAGAAACCTGCCAAG
GAPDH Reverse primer	CCACCACCCTGTTGCTGTAGC

### 2.18. Flow Cytometry

Bone marrow, peripheral blood, and NALF single-cell suspensions were prepared for flow-cytometric analysis. After red blood cell lysis and washing, cells were blocked and stained with fluorochrome-conjugated antibodies. Eosinophils were identified as CCR3 ⁺ Siglec-F⁺ cells using anti-CCR3-PerCP/Cy5.5 and anti-Siglec-F-PE antibodies. CD34 ⁺ progenitor-related cells and CD34 ⁺ CCR3 ⁺ progenitor-related cells were assessed using anti-CD34-PE and anti-CCR3-PerCP/Cy5.5 antibodies. Viability staining, blank controls, isotype controls, and single-stained controls were used for gating and compensation. The same gating strategy was applied across experimental groups within each tissue compartment. Data were analyzed as percentages of the indicated parent population (Number of repetitions = 3).

Compensation was performed using single-stained controls and an automatically generated compensation matrix, followed by visual inspection and manual adjustment when necessary to minimize spectral overlap between PE and PerCP/Cyanine5.5 channels. The same compensation settings and gating strategy were applied consistently across samples within each tissue compartment.

### 2.19. Histological analysis

Mouse heads were decalcified, dehydrated, and embedded in paraffin. Coronal sections (4 μm) were stained with: H&E: Sections were stained with hematoxylin and eosin following standard protocols. PAS: Sections were oxidized, stained with Schiff’s reagent, and counterstained with hematoxylin. Stained sections were examined under a light microscope.

### 2.20. ELISA

Serum levels of eotaxin, IL-5, ECP, and EPO were measured by ELISA according to the manufacturers’ instructions. Before the assay, all reagents were brought to room temperature for 30 min. Standard curves were prepared for eotaxin and IL-5 at 1000, 500, 250, 125, 62.5, 31.25, and 15.6 pg/mL; for ECP at 20, 10, 5, 2.5, 1.25, 0.625, and 0.312 ng/mL; and for EPO at 10, 5, 2.5, 1.25, 0.625, 0.312, and 0.156 ng/mL. Samples were added at 100 μL per well in triplicate and incubated at 37°C for 90 min. After washing, biotinylated detection antibody was added for 60 min, followed by enzyme conjugate incubation for 30 min. After the final wash, TMB substrate was added for color development, the reaction was stopped, and absorbance was measured at 450 nm within 10 min using a microplate reader (Molecular Devices, USA). Concentrations were calculated from the corresponding standard curves.

### 2.21. In Vitro Chemotaxis and Colony Formation Assays

Mononuclear cells from peripheral blood and bone marrow were isolated by Percoll density-gradient centrifugation. Non-adherent mononuclear cells (NAMNCs) were collected after 2 h of adhesion. CD34 ⁺ cells were enriched from NAMNCs using positive selection with PE-conjugated anti-CD34 antibody and anti-PE magnetic beads. The proportion of CD34 ⁺ cells before and after enrichment was assessed by flow cytometry, and cell viability was determined by trypan blue exclusion.

For the chemotaxis assay, CD34 ⁺ -enriched cell fractions were placed in the upper chamber of 24-well transwell plates. Eotaxin (500 ng/mL) or SDF-1α (100 ng/mL) was added to the lower chamber, and migrated cells were counted after 2 h.

For the colony-formation assay, bone marrow NAMNCs were cultured in methylcellulose medium under blank, eotaxin, or IL-5 plus eotaxin stimulation. Colonies were assessed after 14 days of culture following Giemsa staining.

### 2.22. Statistical Analysis

All statistical analyses were performed using GraphPad Prism version 8.0 (GraphPad Software, San Diego, CA, USA) and are presented as mean ± SD unless otherwise specified. Normality and homogeneity of variance were assessed before inferential analyses. For comparisons between two independent groups, an unpaired two-tailed Student’s t-test was used for normally distributed data; otherwise, a Mann–Whitney U test was applied. For comparisons among three or more groups, one-way ANOVA followed by Tukey’s post hoc test was used for normally distributed data with equal variance; otherwise, the Kruskal–Wallis test followed by Dunn’s post hoc test was applied. Correlation analyses were performed using Spearman’s rank correlation coefficient. All tests were two-sided, and p < 0.05 was considered statistically significant.

## 3. Results

Based on our previous work identifying CCR3 as an immune-related gene involved in allergic rhinitis [5,12–14], we next explored transcriptomic, hematopoietic, and functional changes associated with CCR3 deletion in AR models.

### 3.1. Screening of Differentially Expressed Genes and Functional Enrichment Analysis

**Fig 1:**
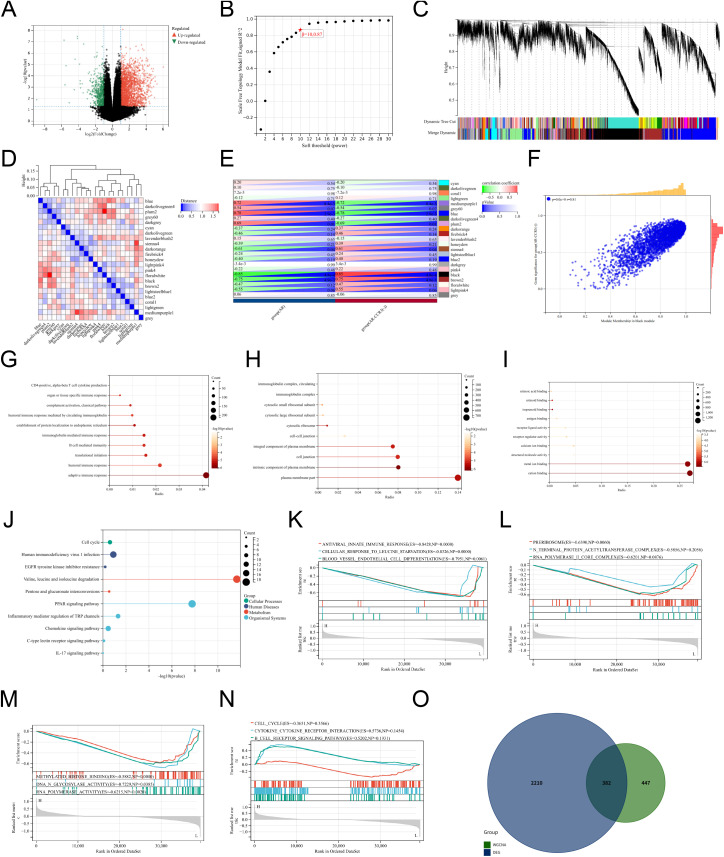
Initial screening of CCR3(-) differentially expressed genes and identification of associated pathways. A Volcano plot of CCR3(-) associated differentially expressed genes (DEGs), with log2FoldChange on the horizontal axis and -log10(P-value) on the vertical axis. Red nodes denote upregulated DEGs, blue nodes represent downregulated DEGs, and grey nodes indicate genes showing no significant differential expression. B Selection of a soft threshold of 10 to achieve a scale-free co-expression network. C Dendrogram branches corresponding to 23 gene modules. D Correlation coefficients between different gene modules. E Correlation coefficients and corresponding p-values between each module and CCR3(-). F Scatter plot of module-specific genes within the black module. G-J GO and KEGG analyses of CCR3(-) differentially expressed genes. K–O GSEA analysis reveals key pathways associated with CCR3(-) differentially expressed genes. O DEG and WGCNA Venn diagram.

By comparing transcriptomic data from CCR3(-) samples with control samples, a total of 2,210 differentially expressed genes were identified (Fig 1A).

To investigate key genes in depth, we selected a soft threshold of 10 to identify gene expression modules. These modules were labelled with distinct colours for descriptive analysis (Fig 1B, 1C, 1D, 1E). It is discernible that certain gene modules exhibit strong synergistic interactions, whilst others demonstrate significant differential expression between the knockout and control cohorts. Examples include the black module (cor = 0.85) and blue module (cor = 0.78). These network modules may play pivotal roles in the biological functions of CCR3(-) allergic rhinitis. For instance, gene expression in the black module highly correlated with baseline levels in CCR3 knockout mice (r = 0.81), encompassing 447 differentially expressed genes.

Overlapping gene sets between knockout and control groups revealed 382 significantly differentially expressed genes following CCR3 knockout (Fig 1O). This novel gene set primarily participates in crucial pathways such as adaptive immune response and plasma membrane part (Fig 1G-1J). Concurrently, GSEA revealed analogous results, demonstrating significant downregulation of immune-related pathways in the knockout group (Fig 1K-1N), including antiviral_innate_immune_response, cell cycle. Collectively, downstream gene alterations following CCR3 knockout in AR mice appear linked to immune activation.

### 3.2. Protein-Protein Interaction Network Construction

**Fig 2:**
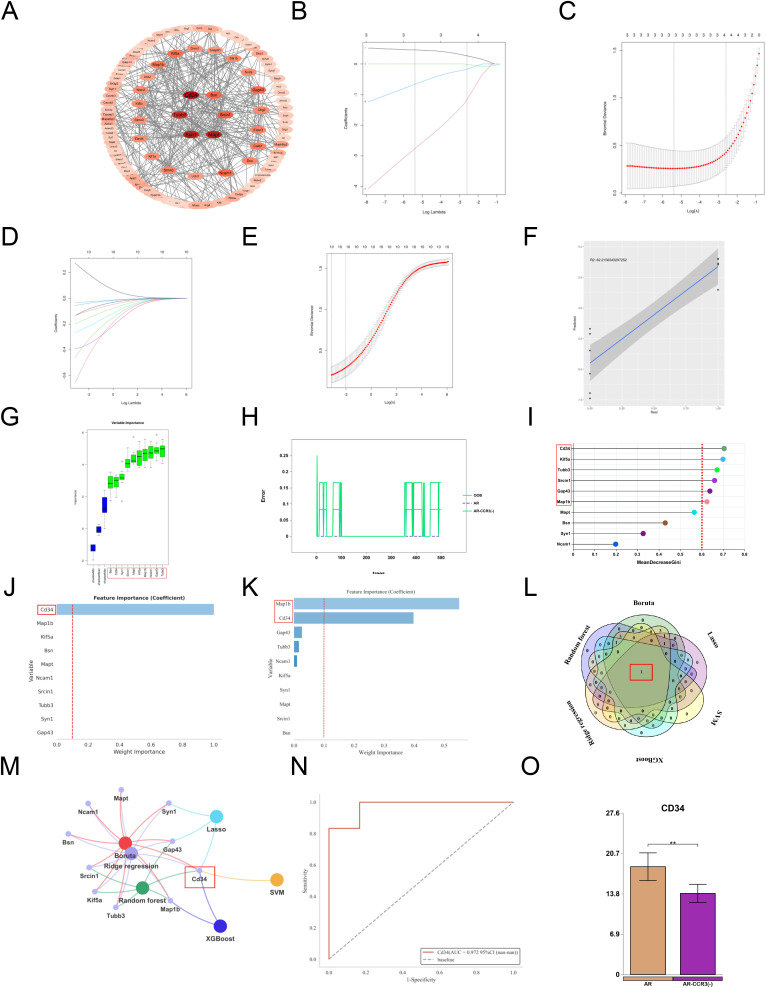
Establishment of PPI networks, machine learning-based evaluation of CCR3(-) hub genes, and validation of hub genes. A Global PPI network of CCR3(-) differentially expressed genes. B–C Biomarker screening using the Lasso model. D–F Biomarker screening using the Ridge regression model. G Biomarker screening using the Boruta model. H–I Random forest algorithm programme displaying error and variable importance plots. J Biomarker screening using the SVM model. K Biomarker screening using the XGBoost model. L Venn diagram illustrating the identification of one hub gene by six machine learning methods. M Network diagram illustrating the six methods employed to identify one candidate diagnostic gene. N ROC curve for CD34. O Bar chart showing differential expression of CD34 between the control group and CCR3(-) group. Data are presented as mean± SD. **P < 0.01.

Subsequently, we constructed a protein-protein interaction (PPI) network comprising the aforementioned 382 differentially expressed genes to elucidate interactions among these functional proteins (Fig 2A). Hub genes within this network were identified using the cytoHubba plugin, ranked by connectivity, and designated as Cd34, Tubb3, Syn1, and Mapt.

### 3.3. Identifying Potential Hub Genes Using Machine Learning

Hub genes were screened using LASSO regression, Boruta, Random Forest, XGBoost, and Ridge regression methods. Fig 2A–2B demonstrate that the LASSO regression algorithm identified three potential candidate biomarkers, while Fig 2D–2F show that the Ridge regression algorithm identified ten potential candidate biomarkers. The Boruta machine learning algorithm is a feature selection method based on the Random Forest algorithm. Its core principle involves comparing the importance of original features with randomly generated shadow features to determine which features correlate with the dependent variable. Fig 2G indicates that the Boruta analysis algorithm identified one potential candidate biomarker. Fig 2H–2I show that the Random Forest analysis algorithm identified six potential candidate biomarkers. Fig 2J indicates that the SVM analysis algorithm identified one potential candidate biomarker. XGBoost (eXtreme Gradient Boosting) is an efficient, flexible, and widely used machine learning algorithm based on the gradient boosting framework. It aims to find the optimal predictive model through multiple iterations. Fig 2K indicates that the XGBoost analysis algorithm identified two potential candidate biomarkers. Subsequently, these six genes were overlaid using a Venn diagram, ultimately yielding a single gene—CD34 (Fig 2L). Fig 2M visualises this process. These analyses identified CD34 as the candidate hub gene most strongly associated with transcriptomic changes observed after CCR3 knockout in AR mice.

### 3.4. Validation of the Hub Gene

CD34 (AUC 0.972, 95% CI NA–NA) (Fig 2N) demonstrated potential diagnostic value for CCR3 knockout based on ROC curve analysis. Concurrently, Fig 2O indicates significant differential expression of CD34 between AR mice and CCR3 knockout mice.

### 3.5. Analysis of Immune Cell Infiltration and Its Relationship with CD34

**Fig 3:**
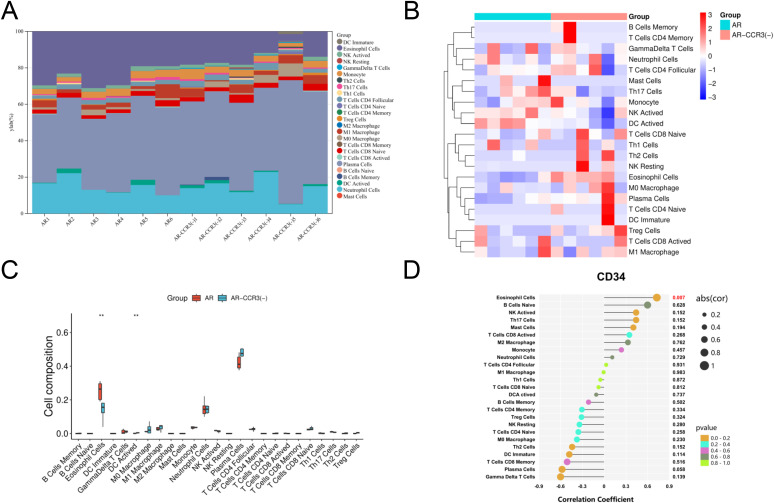
Immunocellular Infiltration Analysis and CD34 Correlation Analysis A–C Bar charts, heatmaps, and box plots of immunocellular proportions in the CCR3(-) group and control group. D Correlation between CD34 and immunocellularity. Data are presented as mean ± SD. **P < 0.01.

We assessed the proportion of immune cell subtypes between the two groups. The findings revealed distinct immunocellular profiles in the CCR3(-) group compared to the control group. Eosinophil-related signals were significantly reduced, whereas activated dendritic cell–related signals were increased in the CCR3(-) group (Fig 3A–3C). CD34 gene expression showed the strongest correlation with eosinophil-related signals (p = 0.007) (Fig 3D). Together, these exploratory findings suggest an association between CCR3 deletion, reduced eosinophil-associated signatures, and altered CD34-associated progenitor abundance in allergic rhinitis. Because CIBERSORT was developed for human transcriptomes, these deconvolution results are presented as exploratory and were validated by flow cytometry (see Section 3.12).

### 3.6. Genotype Identification of Conditionally Knocked-out CCR3 Mice in Bone Marrow Cells

As described in Section 2.11, we generated mice with conditional knockout of the CCR3 gene in bone marrow cells. By breeding Lyz-Cre mice with CCR3fl/fl mice, we obtained CCR3fl/fl&Lyz-Cre and CCR3fl/fl offspring. CCR3fl/fl&Lyz-Cre mice represent our bone marrow cell CCR3 gene conditional knockout mice (CCR3-CKO), while CCR3fl/fl mice represent our littermate wild-type (WT) mice.

#### 3.6.1. Genotype Identification.

As shown in S2 Fig: A band containing only the CCR3 allele fragment (indicating the presence of Loxp sequences, 772 bp) denotes non-knockout mice, designated as WT (wildtype). A band containing both CCR3 and Lyz-Cre recombinase allele fragments (750 bp) denotes knockout mice, designated as CCR3-CKO.

#### 3.6.2. Verification of Knockout Efficiency at mRNA and Protein Levels.

CCR3 knockout efficiency was assessed at both mRNA and protein levels. Following extraction of mouse bone marrow cells, mRNA-level verification revealed significantly reduced CCR3 mRNA expression in the CCR3-CKO group compared to the WT group (S3a Fig). Subsequently, the knockout efficiency was verified at the protein level. Results demonstrated that the protein expression of CCR3 in the CCR3-CKO group was markedly lower than that in the WT group (S3b Fig). This confirms the successful establishment of conditional knockout mice for the CCR3 gene in bone marrow cells.

### 3.7. Comparison of Nasal Symptoms Among Mouse Groups

Consistent with our previous report [13], OVA challenge increased sneezing and nose-scratching frequencies in WT-OVA mice, whereas these symptoms were attenuated in CCR3-CKO-OVA mice. Because the allergic rhinitis model and symptom phenotype have been described previously, these data are presented here only briefly to provide context for the subsequent mechanistic analyses.

### 3.8. Recording body weight changes across groups

**Fig 4:**
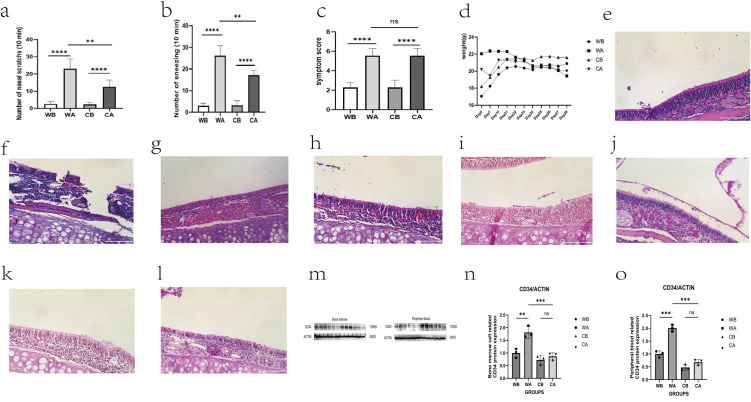
a-c Nasal symptoms in each group of mice. a. Nasal scratching behaviour within 10 minutes after the final nasal challenge; b. Sneezing episodes within 10 minutes after the final nasal challenge; c. Comprehensive symptom scoring for each group of mice. d. Body weight changes during the treatment period for each group of mice. e–h. Pathological changes in the nasal mucosa of each group observed using haematoxylin and eosin (HE) staining: e, WB (WT-Control); f, WA (WT-OVA); g, CB (CKO-Control); h, CA (CKO-OVA). i–l. Glandular hyperplasia and mucus secretion in the nasal mucosa of each group observed using periodic acid-Schiff (PAS) staining: i, WB (WT-Control); j, WA (WT-OVA); k, CB (CKO-Control); l, CA (CKO-OVA). m-o Bone marrow–specific CCR3 deletion was associated with reduced CD34-related mRNA and protein signals in bone marrow and peripheral blood. m, Expression of CD34 protein in bone marrow and peripheral blood of each group; n-o, Expression of CD34 mRNA in bone marrow and peripheral blood of each group; GAPDH served as the control. Note: *P < 0.05, **P < 0.01, ***P < 0.001, ****P < 0.0001, ns indicates P > 0.05, no statistical significance.

Body weights were monitored throughout the treatment period. S6 Table and Fig 4d demonstrate that mice in both the WT-Control and CKO-Control groups initially gained weight before stabilising. In contrast, mice in the WT-OVA and CKO-OVA groups exhibited some weight gain during the sensitisation period but showed a downward trend in body weight during the nasal challenge phase. The WT-OVA group demonstrated a more pronounced weight loss, while the CKO-OVA group exhibited a lesser degree of weight reduction compared to the WT-OVA group. These findings suggest a trend toward reduced weight loss in CCR3-CKO mice during allergic rhinitis induction.

### 3.9. Mouse Nasal Mucosa HE Staining

To investigate the morphological effects of conditional knockout of the CCR3 gene in bone marrow cells on mouse nasal mucosa, histological analysis via HE staining revealed the following: as shown in Fig 4e, 4f, 4g, and 4h, no significant abnormalities were observed in the nasal mucosa of the WT-Control group mice, with no inflammatory cell infiltration. In contrast, the WT-OVA group mice exhibited epithelial detachment and erosion of the nasal mucosa, submucosal swelling, and inflammatory cell infiltration. The nasal mucosa of mice in the CKO-Control group was similar to that in the WT-Control group, showing no obvious abnormalities. In contrast, the nasal mucosa of mice in the CKO-OVA group exhibited a certain degree of damage, but the extent of damage was significantly reduced compared to that in the WT-OVA group. The mucosal epithelial structure was relatively intact, with no obvious oedema, and only a small number of inflammatory cells infiltrated the submucosa. Histological observations suggested that bone marrow–specific CCR3 deletion attenuated nasal mucosal injury and inflammatory cell infiltration.

### 3.10. PAS staining of mouse nasal mucosa

PAS staining was used to qualitatively evaluate mucus production in the nasal mucosa. As shown in Fig 4i–4l, PAS-positive staining was scarcely observed in the WT-Control and CKO-Control groups. In contrast, WT-OVA mice showed increased PAS-positive staining in the nasal mucosa, suggesting enhanced mucus production after OVA challenge. PAS-positive staining appeared reduced in CKO-OVA mice compared with WT-OVA mice.

Because quantitative image analysis of PAS-positive area was not performed in the present study, these findings should be interpreted as qualitative histological observations rather than definitive quantitative evidence of reduced mucus secretion.

### 3.11. Conditional knockout of the CCR3 gene in bone marrow cells is associated with reduced CD34-related mRNA and protein signals in bone marrow and peripheral blood

To investigate the effect of conditional knockout of the CCR3 gene in bone marrow cells on CD34 mRNA and protein expression in allergic rhinitis mice. Following model establishment, bone marrow, peripheral blood, and nasal mucosa samples were collected from each group. CD34 protein levels were measured across four groups. As depicted in Fig 4m, CD34 protein expression in both bone marrow and peripheral blood of WT-OVA group mice was markedly elevated compared to the WT-Control group. Conversely, CD34 protein expression in bone marrow and peripheral blood of CKO-OVA group mice was significantly reduced relative to the WT-OVA group. These findings suggest that OVA challenge is associated with increased CD34-related protein signals in bone marrow and peripheral blood and that bone marrow–specific CCR3 knockout is associated with lower CD34-related signals in these compartments.

qPCR analysis showed that CD34 mRNA levels in bone marrow and peripheral blood were higher in WT-OVA mice than in WT-Control mice, whereas lower levels were observed in CKO-OVA mice than in WT-OVA mice (S7 Table; Fig 4n and 4o). Because CD34 is a classical hematopoietic progenitor marker, these bulk mRNA and protein changes likely reflect altered abundance of CD34 + progenitor cells rather than direct transcriptional regulation of CD34 within individual cells. Due to the low levels of CD34 protein and RNA in the nasal mucosa, reliable intergroup comparison in this tissue was not feasible.

### 3.12. Conditional knockout of the CCR3 gene in bone marrow cells affects the proportion of CD34^+^ progenitor cells, CD34^+^CCR3^+^ progenitor cells, and eosinophils in bone marrow, peripheral blood, and NALF of allergic rhinitis mice

**Fig 5:**
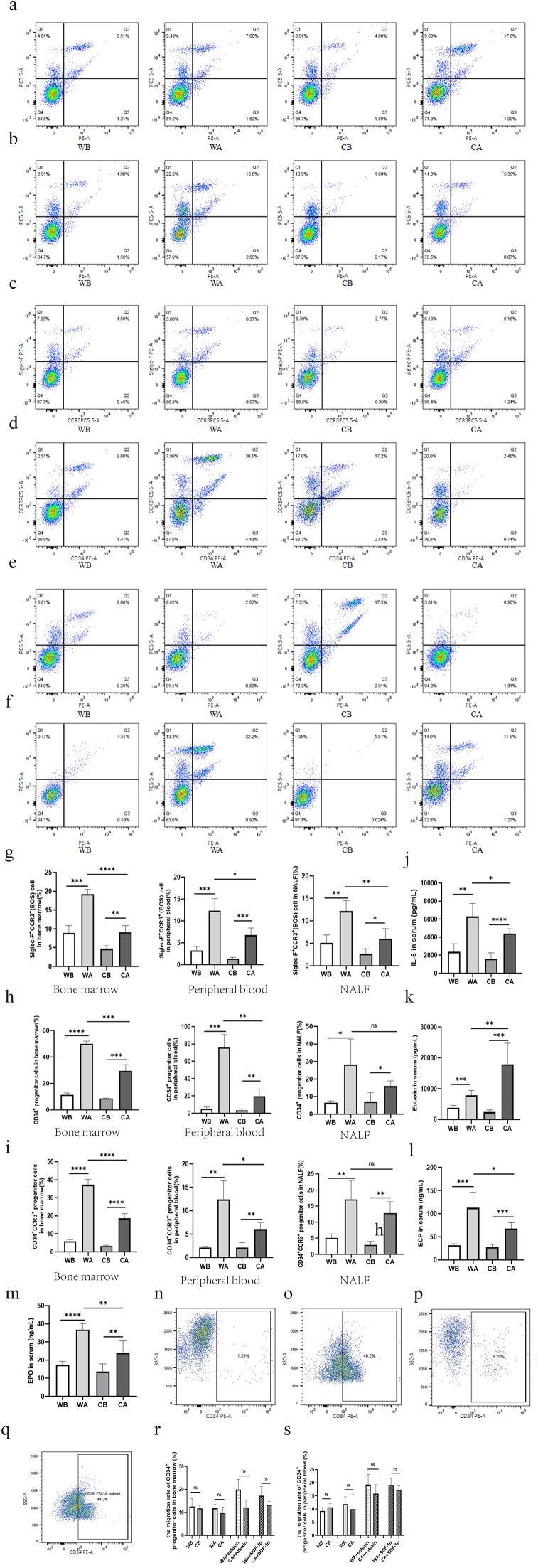
a-i. Effects of conditional knockout of the CCR3 gene in bone marrow cells on the proportions of CD34^+^ progenitor cells, CD34^+^CCR3^+^ progenitor cells, and eosinophils in bone marrow, peripheral blood, and nasal lavage fluid (NALF) of allergic rhinitis mice (N = 3) a. Changes in eosinophil proportion in bone marrow across groups; b. Changes in the proportion of eosinophils in peripheral blood across groups; c. Changes in the proportion of eosinophils in NALF across groups; a-c: PerCP/Cyanine5.5 denotes CCR3, PE denotes Siglec-F; d. Changes in the proportion of CD34^+^ progenitor cells and CD34^+^CCR3^+^ progenitor cells in bone marrow across groups; e. Changes in the proportion of CD34^+^ progenitor cells and CD34^+^CCR3^+^ progenitor cells in peripheral blood across groups; f. Changes in the proportion of CD34^+^ progenitor cells and CD34^+^CCR3^+^ progenitor cells in NALF across groups; d-f: PerCP/Cyanine 5.5 denotes CCR3, PE denotes CD34; g, h, i: statistical graphs showing changes in the proportion of eosinophils, CD34^+^ progenitor cells, and CD34^+^ CCR3^+^ progenitor cells in bone marrow, peripheral blood, and NALF across groups) j-m. Effects of conditional CCR3 gene knockout in bone marrow cells on serum IL-5, eotaxin, ECP, and EPO levels in allergic rhinitis mice (j, k, l, m represent changes in serum IL-5, eotaxin, ECP, and EPO levels in serum from each group) n-q Changes in the proportion of CD34^+^ progenitor cells in mouse bone marrow and peripheral blood after magnetic bead sorting (figures n and o show the proportion of CD34^+^ progenitor cells in the control group and CD34 antibody staining group after magnetic bead sorting of bone marrow cells, respectively; Figures p and q show the CD34^+^ progenitor cell proportions in the control group and CD34 antibody-stained group after magnetic bead separation of peripheral blood, respectively. r-s Bone marrow–specific CCR3 deletion showed a modest, non-significant trend toward reduced in vitro migration of CD34 + progenitor cells (r and s represent changes in the migratory capacity of CD34^+^ progenitor cells in bone marrow and peripheral blood after different treatments, respectively). Note: *P < 0.05, **P < 0.01, ***P < 0.001, ****P < 0.0001, ns indicates P > 0.05, no statistical significance.

Following model establishment, bone marrow cells, peripheral blood, and NALF were collected from each group. Flow cytometry was employed to determine the proportions of eosinophils (CCR3 + Siglec-F^+^), CD34^+^ progenitor cells (CD34^+^), and CD34^+^ CCR3^+^ progenitor cells (CD34^+^ CCR3^+^). Results are presented in Tables 2–4 and Fig 5a–5c, 5g. The proportion of eosinophils in bone marrow, peripheral blood, and NALF was lower in WT-Control and CKO-Control group mice. In contrast, WT-OVA group mice exhibited a significantly elevated proportion of eosinophils in bone marrow, peripheral blood, and NALF, with statistically significant differences. Whereas the proportion of eosinophils in the bone marrow, peripheral blood, and NALF of CKO-OVA group mice decreased markedly compared to WT-OVA group mice. These findings suggest that OVA challenge is associated with eosinophilia and tissue recruitment, whereas bone marrow–specific CCR3 deletion is associated with reduced eosinophil infiltration and circulation.

**Table 2:** Percentage of CD34^+^ progenitor cells, CD34^+^CCR3^+^ progenitor cells, and eosinophils in bone marrow of each mouse group (𝑥 ± 𝑠).

Group	CD34 ^+^ progenitor cells (%)	CD34 ^+^ CCR3 ^+^ progenitor cells (%)	Eosinophils (%)
WT-Control	11.36 ± 1.52	5.8 ± 1.07	8.92 ± 1.97
WT-OVA	49.94 ± 2.1^****^	37.18 ± 3^****^	19.26 ± 1.24^***^
CKO-Control	8.66 ± 0.39^*^	3.31 ± 0.39^**^	4.73 ± 0.77 ^ns^
CKO-OVA	29.49 ± 4.75^***^	18.61 ± 2.67^***^	9.07 ± 1.81 ^ns^

(Note: Compared with WT-Control group: *P < 0.05, **P < 0.01, ***P < 0.001, ****P < 0.0001, ns indicates P > 0.05, no statistical significance)

**Table 3:** Percentage of CD34^+^ progenitor cells, CD34^+^CCR3^+^ progenitor cells, and eosinophils in peripheral blood of mice across groups (𝑥 ± 𝑠).

Group	CD34 ^+^ progenitor cells (%)	CD34 ^+^ CCR3 ^+^ progenitor cells (%)	Eosinophils (%)
WT-Control	5.09 ± 2.24	2.12 ± 0.25	3.26 ± 0.9
WT-OVA	76.01 ± 14.87^****^	12.4 ± 4.02^**^	12.36 ± 2.75^***^
CKO-Control	3.4 ± 1.67 ^ns^	2.09 ± 1.12 ^ns^	1.39 ± 0.27^**^
CKO-OVA	19.67 ± 8.36^*^	6.05 ± 1.45^**^	6.76 ± 1.61^**^

(Note: Compared with WT-Control group: *P < 0.05, **P < 0.01, ***P < 0.001, ****P < 0.0001, ns indicates P > 0.05, no statistical significance)

**Table 4:** Percentages of CD34^+^ progenitor cells, CD34^+^CCR3^+^ progenitor cells, and eosinophils in the NALF of mice across groups (𝑥 ± 𝑠).

Group	CD34 ^+^ progenitor cells (%)	CD34 ^+^ CCR3 ^+^ progenitor cells (%)	Eosinophils (%)
WT-Control	6.5 ± 1.13	5.09 ± 1.22	5.09 ± 1.78
WT-OVA	28.29 ± 14.27^*^	17.09 ± 5.89^**^	12.18 ± 2.35^**^
CKO-Control	7.17 ± 5.27 ^ns^	2.94 ± 1.08^*^	2.66 ± 1.12 ^ns^
CKO-OVA	16.02 ± 3^**^	12.8 ± 3.58^**^	6.01 ± 2.26 ^ns^

(Note: Compared with WT-Control group: *P < 0.05, **P < 0.01, ***P < 0.001, ****P < 0.0001, ns indicates P > 0.05, no statistical significance)

Changes in the proportion of CD34^+^ progenitor cells in bone marrow, peripheral blood, and NALF are shown in Tables 2–4 and Fig 5d-5f, 5h. Mice in the WT-Control and CKO-Control groups exhibited persistently low levels of CD34^+^ progenitor cells across all tissues, whereas the WT-OVA group showed a significant and statistically increase in the proportion of CD34^+^ progenitor cells in all tissues. Although the proportion of CD34^+^ progenitor cells in the NALF of CKO-OVA group mice showed only a partial reduction, the difference was not statistically significant. However, the proportion of CD34^+^ progenitor cells in the bone marrow and peripheral blood of CKO-OVA group mice decreased significantly compared to WT-OVA group mice, and the difference was statistically significant.

As CD34^+^CCR3^+^ progenitor cells constitute the primary cell population responding to eotaxin-mediated chemotaxis and differentiation among CD34^+^ progenitor cells, we investigated whether CD34^+^CCR3^+^ progenitor cells were also present at inflammatory sites. Results, as shown in Tables 2–4 and Fig 5d–5f, 5i, revealed significantly increased proportions of CD34^+^CCR3^+^ cells in the bone marrow, peripheral blood, and NALF of WT-OVA group mice compared to the WT-Control group. Compared with WT-OVA mice, although the proportion of CD34^+^CCR3^+^ progenitor cells in the NALF of CKO-OVA mice was only partially reduced, the levels of CD34^+^CCR3^+^ progenitor cells in the bone marrow and peripheral blood were significantly decreased, with statistically significant differences.

Notably, compared with WT-OVA mice, CKO-OVA mice exhibited a significantly reduced proportion of eosinophils in the NALF (Tables 2–4 and Fig 5c, 5g), whereas CD34^+^ progenitor cells and CD34^+^CCR3 + progenitor cells showed only partial reduction without statistical significance (Tables 2–4 and Fig 5f, 5h-5i). These findings suggest that eosinophil accumulation in the nasal cavity may arise from both local maturation of recruited progenitor populations and trafficking of eosinophils generated in bone marrow. Overall, OVA challenge was associated with increased levels of CD34 + progenitor cells, CD34 + CCR3 + progenitor cells, and eosinophils, whereas conditional CCR3 knockout in bone marrow cells was associated with reduced eosinophil-lineage progenitor abundance and reduced eosinophil infiltration at inflammatory sites in vivo.

### 3.13. Effects of Conditional Knockout of the CCR3 Gene in Bone Marrow Cells on Serum Levels of IL-5, Eotaxin, ECP, and EPO in Mice with Allergic Rhinitis

Extensive research indicates that IL-5 participates in eosinophil differentiation and infiltration at sites of inflammation, whereas eotaxin acts through CCR3. We therefore measured serum IL-5 and eotaxin levels across all groups. As shown in S8 Table and Fig 5j, IL-5 levels were significantly higher in WT-OVA mice than in WT-Control mice, whereas IL-5 levels were markedly lower in CKO-OVA mice than in WT-OVA mice. These findings are consistent with the possibility that reduced IL-5 levels may contribute to altered eosinophil-lineage development in CKO-OVA mice. Eotaxin levels were significantly higher in WT-OVA mice than in WT-Control mice and were further elevated in CKO-OVA mice relative to WT-OVA mice (S8 Table; Fig 5k). This pattern may reflect compensatory feedback within the eotaxin/CCR3 axis rather than enhanced downstream signaling in the absence of CCR3.

To further investigate whether bone marrow cell CCR3 gene knockout affects eosinophil degranulation, we measured serum ECP and EPO concentrations in mice. As shown in S9 Table and Fig 5I-5M, serum ECP and EPO levels were significantly reduced in CKO-OVA mice compared to WT-OVA mice. These findings indicate that conditional knockout of the CCR3 gene in bone marrow cells not only suppresses eosinophil infiltration and circulation in allergic rhinitis mice but also modulates eosinophil degranulation to some extent.

### 3.14. Changes in Proportion and Viability of CD34 + Progenitor Cells Before and After Magnetic Bead Positive Selection

CD34 ⁺ progenitor cells were enriched from mouse bone marrow and peripheral blood mononuclear cells using magnetic bead-based positive selection. As shown in S10 Table and Fig 5n–5q, the proportion of CD34 ⁺ cells increased after selection compared with that before selection, confirming successful CD34 ⁺ cell enrichment. Given the moderate final CD34 ⁺ fraction, these cells were referred to as CD34 ⁺ -enriched fractions rather than highly purified CD34 ⁺ cells. As shown in S11 Table, cell viability did not differ significantly before and after selection in either bone marrow or peripheral blood samples, indicating that the enrichment procedure preserved cell viabilit**y.**

### 3.15. Bone marrow–specific CCR3 deletion shows a modest, non-significant trend toward reduced in vitro migration of CD34 + progenitor cells

This experiment further employed chemotaxis assays to determine whether conditional knockout of the CCR3 gene in bone marrow cells modulates the in vitro migration of CD34^+^ progenitor cells. Results, as shown in Table 5, Table 6 and Fig 5r-s, indicate that without treatment with eotaxin or stromal cell-derived factor-1α (SDF-1α), CD34^+^ progenitor cell migration capacity was low in both bone marrow and peripheral blood, regardless of whether the bone marrow cell CCR3 gene was conditionally knocked out. Furthermore, OVA stimulation was found to fail to enhance CD34^+^ progenitor cell migration capacity. In contrast, following exposure to eotaxin or SDF-1α, CD34^+^ progenitor cells in the bone marrow and peripheral blood of WT-OVA group mice exhibited a partial increase in migration rate. Compared to the WT-OVA group, the migration rate of CD34^+^ cells in the CKO-OVA group was reduced, though neither difference was statistically significant. These findings suggest that the eotaxin/CCR3 axis may exert only a modest effect on isolated progenitor chemotaxis under the present experimental conditions. Additional pathways are likely required to support robust migratory responses.

**Table 5:** Effects of conditional knockout of the CCR3 gene in bone marrow cells on chemotaxis of bone marrow CD34^+^ progenitor cells (𝑥 ± 𝑠).

Group	Saline	OVA	OVA+eotaxin	OVA + SDF-1α
WildType	12.59 ± 3.45	11.78 ± 1.74 ^ns^	19.93 ± 4.49 ^ns^	17.25 ± 4.11 ^ns^
CCR3-KO	11.72 ± 1.41 ^ns^	9.95 ± 2.35 ^ns^	12.14 ± 3.15 ^ns^	13.29 ± 1.46 ^ns^

(Note: Compared with physiological saline treatment in the WildType group: *P < 0.05, **P < 0.01, ***P < 0.001, ****P < 0.0001, ns indicates P > 0.05, no statistical significance)

**Table 6:** Effect of conditional knockout of the CCR3 gene in bone marrow cells on the chemotactic response of peripheral blood CD34^+^ progenitor cells (𝑥 ± 𝑠).

Group	Saline	OVA	OVA+eotaxin	OVA + SDF-1α
WildType	9.23 ± 1.33	11.83 ± 2.85 ^ns^	19.29 ± 3.93	19.14 ± 2.54
CCR3-KO	10.65 ± 1.39 ^ns^	9.91 ± 5.6 ^ns^	15.88 ± 3.43	17.27 ± 1.85

(Note: Compared with physiological saline treatment in the WildType group: *P < 0.05, **P < 0.01, ***P < 0.001, ****P < 0.0001, ns indicates P > 0.05, no statistical significance)

### 3.16. Bone marrow cell-specific CCR3 deletion attenuates IL-5/eotaxin-associated colony formation from NAMNCs

**Fig 6:**
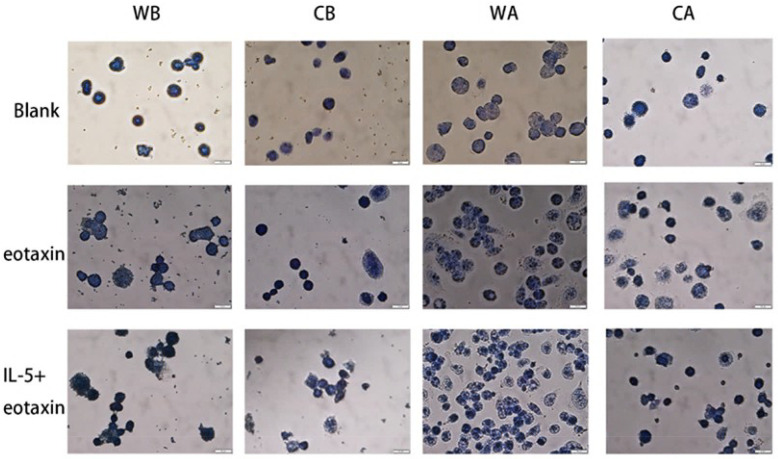
Bone marrow-specific CCR3 deletion attenuates IL-5/eotaxin-associated colony formation of NAMNCs.

Bone marrow non-adherent mononuclear cells (NAMNCs) from each group were cultured for 14 days in methylcellulose medium under blank, eotaxin, or IL-5 plus eotaxin stimulation. As shown in Fig 6, eotaxin stimulation increased colony formation, and IL-5 plus eotaxin further enhanced eosinophil-lineage–associated colony formation. Compared with the WT-OVA group, NAMNCs from the CKO-OVA group showed reduced colony-forming capacity.

These findings suggest that eotaxin and IL-5 may cooperate to promote eosinophil-lineage–associated colony formation from bone marrow NAMNCs during allergic inflammation, whereas this response appeared to be attenuated by bone marrow cell-specific CCR3 deletion. Because this assay was performed using NAMNCs rather than highly purified CD34 ⁺ progenitor cells, the results should be interpreted as supportive functional evidence rather than direct proof of CCR3-dependent CD34 ⁺ progenitor differentiation.

## 4. Discussion

The biological effects of chemokines are initiated through their binding to G protein-coupled receptors present on cell membranes, a process that directly regulates the trafficking of leukocytes to regions of inflammation [34,35]. Elevated chemokine expression is closely linked to the development of inflammatory conditions like asthma and atherosclerosis. Several classes of chemokine receptors have been documented in humans, including CCR, CXCR, CX3CR, and XCR families [36]. Stimulation of these receptors on leukocytes induces downstream signaling events that strongly drive chemotaxis, leading to selective accumulation of immune cells at inflamed sites. Among these receptors, CCR3 appears to have a particularly important function in allergic inflammation [15,37]. The cellular infiltrate characteristic of allergic diseases is mainly composed of eosinophils, Th2 cells, and mast cells—each expressing CCR3 on their surfaces. Animal studies further reveal substantial increases in eotaxin, a ligand for CCR3, in allergic airway models, with clear evidence supporting the role of this receptor in guiding eosinophil recruitment during inflammatory responses [38]. Structurally, CCR3 is a seven-transmembrane domain receptor coupled to G proteins, with its highest expression observed on eosinophils, where it serves as the dominant surface receptor. Multiple investigations confirm that CCR3 contributes significantly to various inflammatory disorders via eosinophil-mediated mechanisms. To illustrate, work by Park and colleagues indicated that glutathione S-transferase administration lowers both CCR3 and eotaxin levels in a murine model of allergic dermatitis, which correlated with decreased eosinophil numbers and amelioration of clinical signs such as erythema and eczema [39]. In a separate study, Filippone et al. found that CCR3 antagonism improved gastrointestinal dysfunction in experimental colitis by preventing eosinophil accumulation at inflamed intestinal sites [40]. Moreover, eosinophils were nearly undetectable in colonic tissues obtained from CCR3-knockout mice [41]. Eotaxin binding to CCR3 activates several intracellular signaling cascades, including the MAPK (ERK1/2, p38) and PI3K/AKT pathways [42,43]. These signals collectively promote eosinophil priming, directional migration, cellular activation, and degranulation [44,45]. Eosinophils contribute critically to the late-phase allergic reaction in rhinitis [46], however, the extent to which eotaxin modulates AR pathogenesis by potentiating eosinophil activity through CCR3 was not fully established. Our data reveal a pronounced elevation in eosinophil numbers within the bone marrow, peripheral blood, and NALF of AR mice. By contrast, mice with bone marrow–restricted deletion of CCR3 showed significantly fewer eosinophils in these compartments, a result further supported by immune cell infiltration analyses. These observations support an association between bone marrow cell-specific CCR3 deletion and reduced eosinophil-related cell abundance in the analyzed compartments.

The late-phase reaction in AR features dynamic interactions among Th2 cells, eosinophils, basophils, and neutrophils. Eosinophils occupy an important position in this allergic network. Upon encountering antigen, eosinophils generate a range of mediators—including EPO, MBP, ECP, and END—in addition to chemotactic factors such as IL-5 and eotaxin. These substances participate in airway hyperreactivity, loss of epithelial integrity, and structural remodeling of tissues [47–51]. Degranulation of eosinophils within affected tissues is widely regarded as a major driver of pathology in chronic eosinophil-linked diseases. Studies using degranulation assessment models indicate that nearly all eosinophils present in AR nasal mucosa show clear signs of granule release [52]. A substantial body of research supports the notion that eosinophils and their secreted products have central roles in AR pathophysiology [53–56]. Our earlier report [57] indicated that suppressing CCR3 expression reduces the production, migration, and invasiveness of eosinophils in AR mice, effectively diminishing their functional capacity. Other investigators have documented that therapeutic targeting of CCR3 substantially limits eosinophil influx into the lungs after allergen provocation, which coincides with decreased airway hyperresponsiveness and mucus hypersecretion [58]. In the current work, conditional CCR3 knockout in bone marrow cells led to a pronounced reduction in serum concentrations of IL-5, ECP, and EPO in AR mice. Interestingly, this genetic manipulation resulted in elevated systemic eotaxin levels. These outcomes suggest that CCR3 loss not only restrains eosinophil infiltration and systemic circulation but also partly influences eosinophil degranulation. One plausible explanation involves a negative feedback relationship within the eotaxin/CCR3 system: reduced CCR3 surface expression on effector cells caused by conditional knockout may trigger compensatory rises in endogenous eotaxin synthesis.

Eosinophils are granulocytic cells originating from CD34^+^ progenitor cells [59,60]. Antigen exposure induces CD34^+^ progenitors residing in the bone marrow to proliferate and mature into eosinophils, which then enter the circulation and migrate to the airways [8]. Although bone marrow hematopoiesis can mount a systemic response to allergens—promoting eosinophil maturation, production, and release—emerging findings indicate that allergen-activated CD34^+^ progenitors can also travel to the airways and differentiate into eosinophils directly within inflamed tissues, a process referred to as in situ hematopoiesis. This local differentiation mechanism depends on the presence of IL-5 and eotaxin [61,62]. Notably, relevant studies have elegantly demonstrated the direct effect of eotaxin on promoting the differentiation of CD34 ⁺ progenitor cells into eosinophils, potentially independent of IL-3, IL-5, and GM-CSF [63]. For example, Judah et al. [64] reported elevated numbers of CD34^+^ progenitors in peripheral blood and bronchial mucosal samples from chronic bronchitis patients, with their abundance positively associated with eosinophil counts. Additional reports have shown that the eotaxin/CCR3 axis participates in the local conversion of CD34^+^ cells into eosinophils in the airways following allergen challenge [65–67]. These collective findings suggest that increased CCR3 expression on bone marrow CD34^+^ progenitors could facilitate their eotaxin-dependent release into peripheral blood. Once in the circulation, under the influence of local inflammatory mediators including IL-5 and eotaxin, these cells may first transition into CD34^+^CCR3^+^ progenitor cells before final maturation into eosinophils. These locally differentiated cells may then cooperate with bone marrow–derived eosinophils to sustain allergic airway inflammation. Our approach, combining bioinformatics and machine learning, identified CD34 as a candidate gene associated with CCR3 deletion. Because CD34 is a classical hematopoietic progenitor marker, the observed changes in bulk CD34 mRNA and protein signals should be interpreted primarily as changes in CD34 ⁺ progenitor-related cell abundance rather than direct transcriptional regulation of CD34 expression within individual cells. Flow-cytometry analysis further showed altered proportions of CD34 ⁺ progenitors and CD34 ⁺ CCR3 ⁺ progenitor-related cells in bone marrow and peripheral blood after OVA challenge and CCR3 deletion. In NALF, eosinophils were reduced in CKO-OVA mice compared with WT-OVA mice, whereas CD34⁺ and CD34 ⁺ CCR3 ⁺ progenitor-related populations showed only partial reductions. These findings suggest that eosinophil-related inflammation in AR may involve both systemic eosinophil-lineage output and local progenitor-cell dynamics. However, the present data do not establish whether CCR3 directly controls CD34 expression or eosinophil-lineage commitment at the single-cell level.

Previous studies have established that the interaction between SDF-1α and its cognate receptor CXCR4 facilitates mobilization and migration of CD34^+^ progenitors into peripheral blood [68,69]. Other chemokine receptors detected in early hematopoietic niches include CCR3, CCR9, CCR5, CXCR1, CXCR2, and CXCR3 [70,71]. Nevertheless, their specific roles in CD34^+^ progenitor migration remain poorly understood. Here, we observed that AR was associated with enhanced migration-related behavior of CD34 + progenitors from bone marrow to inflammatory loci via peripheral blood, together with a shift toward the eosinophil lineage. To better define the functional contribution of CCR3 to CD34^+^ progenitor migration and differentiation, we conducted in vitro assays using isolated CD34^+^ progenitors. Chemotaxis experiments indicated that eotaxin exposure moderately increased the migratory ability of allergen-stimulated CD34^+^ progenitors. By comparison, CD34^+^ progenitors from bone marrow–specific CCR3-knockout mice showed a partial reduction in migration following eotaxin stimulation. These results imply that the eotaxin/CCR3 pathway exerts a modest positive effect on CD34^+^ progenitor migration. Because the post-selection CD34 ⁺ fraction indicated enrichment rather than complete purification, functional assays based on these cells should be interpreted as analyses of CD34 ⁺ -enriched fractions rather than highly purified CD34 ⁺ progenitors. It is possible that this effect depends on cooperative interactions with other signaling pathways to achieve full migratory responsiveness, suggesting that the mechanisms governing CD34^+^ progenitor migration deserve further study. In colony-formation assays performed with mouse bone marrow NAMNCs, eotaxin in combination with IL-5 promoted eosinophil-lineage–associated colony formation. Genetic deletion of CCR3 in bone marrow cells partially reduced the colony-forming ability of NAMNCs. This supports the possibility that eotaxin and IL-5 cooperate to regulate eosinophil-lineage–associated colony formation, but it should not be interpreted as direct proof of CD34 ⁺ progenitor differentiation.

Using Cre/loxP technology, this study selectively disrupted CCR3 in bone marrow-derived cells and established an OVA-induced AR model. Local eosinophilic inflammation was supported by NALF flow-cytometry findings and nasal histological observations. Overall, the present data suggest that bone marrow cell-specific CCR3 deletion is associated with reduced eosinophil-lineage–related progenitor abundance and decreased eosinophil-associated inflammatory responses in vivo. However, these findings should not be interpreted as direct evidence that CCR3 independently determines CD34 ⁺ progenitor differentiation or eosinophil-lineage commitment.

It is important to acknowledge the rationale and potential limitations of our experimental models. While classical AR models frequently utilize Th2-prone BALB/c mice, the bone marrow-specific CCR3 conditional knockout model in the current study was intrinsically established on a C57BL/6 background^5^. Despite being more Th1-prone, our data robustly demonstrate that OVA-induced eosinophilic inflammation was successfully established, providing a viable platform to study CCR3 mechanisms. Additionally, our bioinformatic analysis utilizing the human-optimized CIBERSORT LM22 matrix to estimate murine immune infiltration relied on ortholog gene mapping. While widely accepted, it may introduce interspecies biases.While widely accepted, it may introduce interspecies biases. Furthermore, regarding our in vitro assays, while we utilized 500 ng/mL of eotaxin to establish a robust chemotactic gradient, we did not evaluate lower concentrations; thus, potential chemorepulsion or receptor desensitization effects cannot be entirely ruled out.

Regarding mechanism, the bulk RNA-seq data suggesting CD34 as a candidate gene associated with CCR3 deletion likely reflects an increase in the overall abundance and frequency of CD34 ⁺ progenitor cells in the bone marrow rather than direct transcriptional upregulation of the CD34 gene within individual cells. We also recognize that IL-5 remains a master regulator of eosinophil differentiation. Because we did not perform in vivo IL-5 neutralization or exogenous IL-5 rescue in CCR3-CKO mice, the relative contributions of direct CCR3 signaling versus IL-5-dependent pathways could not be completely separated. Combined blockade experiments will be an essential direction for our future research.

In addition, eosinophils were not separately quantified in nasal mucosal sections. Local eosinophilic inflammation was evaluated using NALF flow-cytometry readouts together with nasal histological observations, which should be interpreted as supportive local inflammatory evidence rather than direct quantitative enumeration of eosinophils within nasal mucosal tissue.

Finally, the seemingly paradoxical observation of elevated systemic eotaxin levels alongside reduced eosinophil tissue infiltration in CCR3-CKO mice can be explained by the loss of the “chemokine receptor sink” effect [72]. In wild-type conditions, functional CCR3 receptors bind, internalize, and clear eotaxin from the microenvironment. The deletion of CCR3 abrogates this clearance, leading to extracellular eotaxin accumulation in the serum. Simultaneously, lacking the requisite receptor, these cells cannot respond to the intense eotaxin gradient to migrate into the mucosa, thus resolving the apparent contradiction.

## 5. Conclusion

In summary, bone marrow–specific CCR3 deletion was associated with reduced eosinophilic inflammation and altered CD34 ⁺ progenitor-related signals in allergic rhinitis. Our findings suggest that CCR3 may contribute to eosinophil-lineage commitment and progenitor cell dynamics during allergic inflammation. However, the present data more strongly support altered progenitor abundance and eosinophil-related responses than direct regulation of CD34 expression at the single-cell level. Further studies using single-cell and lineage-tracing approaches are needed to clarify the precise mechanisms involved.

## Supporting information

S1 Table Reverse transcription reaction mix for RNA (genomic DNA removal). (DOCX)

S2 Table Reverse transcription reaction mix for cDNA synthesis. (DOCX)

S3 Table qPCR Reaction System. (DOCX)

S4 Table qPCR Amplification Conditions. (DOCX)

S5 Table Symptom Scores and Number of Nose-Scratching and Sneezing Episodes within 10 Minutes Post-Final Stimulation in Mice (𝑥 ± 𝑠). (DOCX)

S6 Table Body weight changes (g) during drug administration in each group. (DOCX)

S7 Table Levels of CD34 mRNA in Bone Marrow and Peripheral Blood of Mice in Each Group (𝑥 ± 𝑠). (DOCX)

S8 Table Serum concentrations of IL-5 and eotaxin in mice across groups (𝑥 ± 𝑠). (DOCX)

S9 Table Serum concentrations of ECP and EPO in mice across groups (𝑥 ± 𝑠). (DOCX)

S10 Table CD34 + progenitor cell purity (𝑥 ± 𝑠) in bone marrow and peripheral blood before and after magnetic bead-based selection. (DOCX)

S11 Table Cell viability (𝑥 ± 𝑠) in bone marrow and peripheral blood before and after magnetic bead-positive sorting. (DOCX)

S1 Fig Mouse Construction Flowchart. (DOCX)

S2 Fig Mouse Genotype Identification. (DOCX)

S3 Fig Validation of knockout efficiency at RNA and protein levels(a. qPCR detection of CCR3 mRNA expression levels in mouse bone marrow cells; b. Western blot detection of CCR3 protein expression levels in mouse bone marrow cells. Note: *P < 0.05, **P < 0.01, ***P < 0.001, ****P < 0.0001, ns indicates P > 0.05, no statistical significance).
(DOCX)
