# Supplementary material for: Bone marrow CCR3 dictates eosinophil lineage commitment of CD34⁺ progenitors to orchestrate allergic rhinitis: A composite study
Source: PLoS One. 2026 Jun 22;21(6):e0351726. doi: 10.1371/journal.pone.0351726 (PMC13286145; doi:10.1371/journal.pone.0351726)
Supplement: S1 Table — (DOCX) [file pone.0351726.s001.docx]

Supplementary Table 1: Reverse transcription reaction mix for RNA (genomic DNA

| 10x gDNA plus remover mix | 1μl |
| --- | --- |
| RNA Template | 0.01-1 µg |
| DEPC-ddH_2_O | Make up to 10μl |
