# Supplementary material for: Bone marrow CCR3 dictates eosinophil lineage commitment of CD34⁺ progenitors to orchestrate allergic rhinitis: A composite study
Source: PLoS One. 2026 Jun 22;21(6):e0351726. doi: 10.1371/journal.pone.0351726 (PMC13286145; doi:10.1371/journal.pone.0351726)
Supplement: S2 Table — (DOCX) [file pone.0351726.s002.docx]

Supplementary Table2: Reverse transcription reaction mix for cDNA synthesis

| 5x M5 RT Super plus Mix | 4μl |
| --- | --- |
| DEPC-ddH_2_O | 6μl |
| Step (a) Reaction Mixture | 10μl |
| total | 20μl |
