# Supplementary material for: Bone marrow CCR3 dictates eosinophil lineage commitment of CD34⁺ progenitors to orchestrate allergic rhinitis: A composite study
Source: PLoS One. 2026 Jun 22;21(6):e0351726. doi: 10.1371/journal.pone.0351726 (PMC13286145; doi:10.1371/journal.pone.0351726)
Supplement: S3 Table — (DOCX) [file pone.0351726.s003.docx]

Supplementary Table 3 qPCR Reaction System: qPCR Reaction System

| Reagent | Volume（μl） |
| --- | --- |
| 2x SYBR | 10 |
| Upstream Primer（10 nM） | 1 |
| Downstream Primer（10 nM） | 1 |
| ROX Reference Dye II (50x) | 0.4 |
| cDNA | 2 |
| ddH_2_O | 5.6 |
| total | 20 |
