# Supplementary material for: Bone marrow CCR3 dictates eosinophil lineage commitment of CD34⁺ progenitors to orchestrate allergic rhinitis: A composite study
Source: PLoS One. 2026 Jun 22;21(6):e0351726. doi: 10.1371/journal.pone.0351726 (PMC13286145; doi:10.1371/journal.pone.0351726)
Supplement: S4 Table — (DOCX) [file pone.0351726.s004.docx]

Supplementary Table 4 qPCR Amplification Conditions: qPCR Amplification Conditions

| Step | Temp℃ | Time | Note |
| --- | --- | --- | --- |
| 1 | 95 | 30sec |  |
| 2 | 95 | 5sec |  |
| 3 | 60 | 34sec | Repeat step 2-3 for 40 cycle |
| 4 | 95 | 15sec |  |
| 5 | 60 | 1min | Melt Curve step 4-5 |
