# Supplementary material for: Bone marrow CCR3 dictates eosinophil lineage commitment of CD34⁺ progenitors to orchestrate allergic rhinitis: A composite study
Source: PLoS One. 2026 Jun 22;21(6):e0351726. doi: 10.1371/journal.pone.0351726 (PMC13286145; doi:10.1371/journal.pone.0351726)
Supplement: S5 Table — (DOCX) [file pone.0351726.s005.docx]

Supplementary Table 5: Symptom Scores and Number of Nose-Scratching and Sneezing Episodes within 10 Minutes Post-Final Stimulation in Mice (𝑥̅± 𝑠)

| Group | Symptom Score (points) | Nose-Scratching (times) | Sneezing (times) |
| --- | --- | --- | --- |
| WT-Control | 2.29±0.49 | 2.57±1.4 | 3±1.15 |
| WT-OVA | 5.56±0.73^****^ | 23±5.69^****^ | 26.25±4.57^****^ |
| CKO-Control | 2.29±0.76 ^ns^ | 2.43±1.13 ^ns^ | 3.14±2.19 ^ns^ |
| CKO-OVA | 5.56±0.73^****^ | 13.71±2.5^****^ | 17.2±2.17^****^ |

(Note: Compared with WT-Control group: *P＜0.05, **P＜0.01, ***P＜0.001, ****P＜0.0001, ns indicates P>0.05, no statistical significance )
