# Supplementary material for: Bone marrow CCR3 dictates eosinophil lineage commitment of CD34⁺ progenitors to orchestrate allergic rhinitis: A composite study
Source: PLoS One. 2026 Jun 22;21(6):e0351726. doi: 10.1371/journal.pone.0351726 (PMC13286145; doi:10.1371/journal.pone.0351726)
Supplement: S6 Table — (DOCX) [file pone.0351726.s006.docx]

Supplementary Table 6: Body weight changes (g) during drug administration in each group

| Group | WT-Control | WT-OVA | CKO-Control | CKO-OVA |
| --- | --- | --- | --- | --- |
| Day0 | 17.05±2.7 | 22.05±1.56 | 18.21±2.67 | 20.2±2.72 |
| Day7 | 18.24±2.66 | 22.37±1.43 | 19.26±3.57 | 19.47±5.24 |
| Day14 | 19.63±1.9 | 22.32±1.83 | 20.47±2.8 | 21.28±2.51 |
| Day21 | 20.4±2.2 | 22.3±1.91 | 21.44±2.27 | 21.32±2.82 |
| Day22 | 20.56±2.33 | 21.63±1.84 | 21.4±2.34 | 21.08±2.93 |
| Day23 | 20.36±2.31 | 21.22±1.63 | 21.51±2.35 | 21.2±2.92 |
| Day24 | 20.18±2.52 | 20.52±1.81 | 21.26±2.39 | 20.63±2.78 |
| Day25 | 20.61±2.47 | 20.47±1.96 | 21.71±2.28 | 20.7±2.91 |
| Day26 | 20.75±2.45 | 20.43±2.22 | 21.74±2.39 | 20.58±2.72 |
| Day27 | 20.58±2.59 | 20.08±2.12 | 21.63±2.5 | 20.13±2.94 |
| Day28 | 20.89±2.67 | 19.43±1.83 | 21.61±2.51 | 20.02±3.01 |
