# Supplementary material for: Bone marrow CCR3 dictates eosinophil lineage commitment of CD34⁺ progenitors to orchestrate allergic rhinitis: A composite study
Source: PLoS One. 2026 Jun 22;21(6):e0351726. doi: 10.1371/journal.pone.0351726 (PMC13286145; doi:10.1371/journal.pone.0351726)
Supplement: S7 Table — (DOCX) [file pone.0351726.s007.docx]

Supplementary Table 7: Levels of CD34 mRNA in Bone Marrow and Peripheral Blood of Mice in Each Group (𝑥̅± 𝑠)

| Group | Bone Marrow | Peripheral Blood |
| --- | --- | --- |
| WT-Control | 1.02±0.24 | 1.02±0.22 |
| WT-OVA | 3.37±0.77^****^ | 3.72±0.77^****^ |
| CKO-Control | 0.42±0.45^*^ | 0.57±0.43 ^ns^ |
| CKO-OVA | 1.05±0.74 ^ns^ | 0.62±0.3^*^ |

(Note: Compared with WT-Control group: *P＜0.05, **P＜0.01, ***P＜0.001, ****P＜0.0001, ns indicates P>0.05, no statistical significance )
