# Supplementary material for: Bone marrow CCR3 dictates eosinophil lineage commitment of CD34⁺ progenitors to orchestrate allergic rhinitis: A composite study
Source: PLoS One. 2026 Jun 22;21(6):e0351726. doi: 10.1371/journal.pone.0351726 (PMC13286145; doi:10.1371/journal.pone.0351726)
Supplement: S8 Table — (DOCX) [file pone.0351726.s008.docx]

Supplementary Table 8: Serum concentrations of IL-5 and eotaxin in mice across groups (𝑥̅± 𝑠)

| Group | IL-5 (pg/ml） | eotaxin(pg/ml) |
| --- | --- | --- |
| WT-Control | 2106.77±910.84 | 3871.7±745.7 |
| WT-OVA | 6299.74±1233.36^**^ | 7831.9±1614.75^***^ |
| CKO-Control | 1576.31±757.72 ^ns^ | 2413.75±712.34^**^ |
| CKO-OVA | 4392.08±366.68^**^ | 17854.58±6908.8^***^ |

(Note: Compared with WT-Control group: *P＜0.05, **P＜0.01, ***P＜0.001, ****P＜0.0001, ns indicates P>0.05, no statistical significance )
