# Supplementary material for: Bone marrow CCR3 dictates eosinophil lineage commitment of CD34⁺ progenitors to orchestrate allergic rhinitis: A composite study
Source: PLoS One. 2026 Jun 22;21(6):e0351726. doi: 10.1371/journal.pone.0351726 (PMC13286145; doi:10.1371/journal.pone.0351726)
Supplement: S9 Table — (DOCX) [file pone.0351726.s009.docx]

Supplementary Table 9: Serum concentrations of ECP and EPO in mice across groups (𝑥̅± 𝑠)

| Group | ECP（ng/ml） | EPO(ng/ml） |
| --- | --- | --- |
| WT-Control | 31.89±3.22 | 17.39±1.96 |
| WT-OVA | 112.7±33.41^***^ | 36.74±3.43^****^ |
| CKO-Control | 27.54±6.26 ^ns^ | 13.56±4.37 ^ns^ |
| CKO-OVA | 67.88±12.89^***^ | 23.96±6.71^*^ |

(Note: Compared with WT-Control group: *P＜0.05, **P＜0.01, ***P＜0.001, ****P＜0.0001, ns indicates P>0.05, no statistical significance )
