# Supplementary material for: Bone marrow CCR3 dictates eosinophil lineage commitment of CD34⁺ progenitors to orchestrate allergic rhinitis: A composite study
Source: PLoS One. 2026 Jun 22;21(6):e0351726. doi: 10.1371/journal.pone.0351726 (PMC13286145; doi:10.1371/journal.pone.0351726)
Supplement: S10 Table — (DOCX) [file pone.0351726.s010.docx]

Supplementary Table 10: CD34^+^ progenitor cell purity (𝑥̅± 𝑠) in bone marrow and peripheral blood before and after magnetic bead-based selection

| Sample | Purity before selection | Purity after selection |
| --- | --- | --- |
| Bone Marrow | 8.58±0.65 | 57.36±5.49^****^ |
| Peripheral Blood | 10.11±5.51 | 59.71±6.81^****^ |

(Note: Compared with WT-Control group: *P＜0.05, **P＜0.01, ***P＜0.001, ****P＜0.0001, ns indicates P>0.05, no statistical significance )
