# Supplementary material for: Bone marrow CCR3 dictates eosinophil lineage commitment of CD34⁺ progenitors to orchestrate allergic rhinitis: A composite study
Source: PLoS One. 2026 Jun 22;21(6):e0351726. doi: 10.1371/journal.pone.0351726 (PMC13286145; doi:10.1371/journal.pone.0351726)
Supplement: S11 Table — (DOCX) [file pone.0351726.s011.docx]

Supplementary Table 11: Cell viability (𝑥̅± 𝑠) in bone marrow and peripheral blood before and after magnetic bead-positive sorting

| Sample | Purity before selection | Purity after selection |
| --- | --- | --- |
| Bone | 93.67±2.31 | 89.83±1.76 ^ns^ |
| Peripheral Blood | 96±1 | 91.67±2.52 ^ns^ |

(Note: Compared with WT-Control group: *P＜0.05, **P＜0.01, ***P＜0.001, ****P＜0.0001, ns indicates P>0.05, no statistical significance )
