# Supplementary figures and images for: Bone marrow CCR3 dictates eosinophil lineage commitment of CD34⁺ progenitors to orchestrate allergic rhinitis: A composite study
Source: PLoS One. 2026 Jun 22;21(6):e0351726. doi: 10.1371/journal.pone.0351726 (PMC13286145; doi:10.1371/journal.pone.0351726)

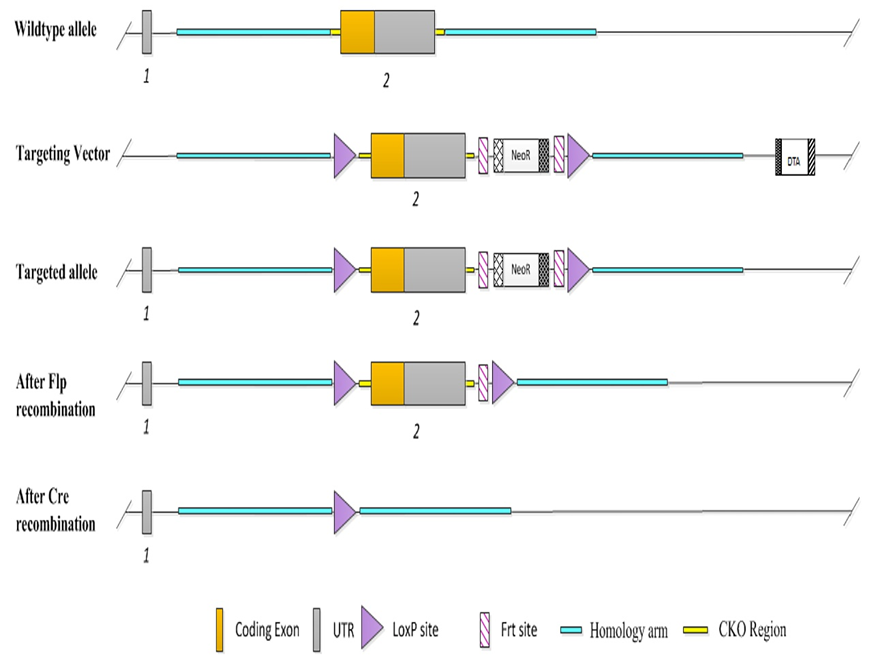


Supplementary Figure 1: Mouse Construction Flowchart

Supplement: S1 Fig — (DOCX) [file pone.0351726.s012.docx]

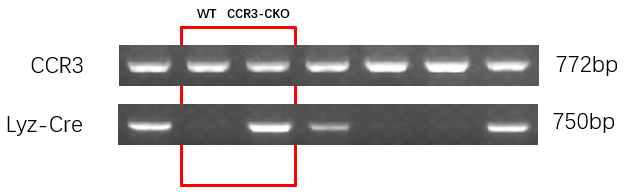


Supplementary Figure 2: Mouse Genotype Identification

Supplement: S2 Fig — (DOCX) [file pone.0351726.s013.docx]
