# Supplementary material for: Bone marrow CCR3 dictates eosinophil lineage commitment of CD34⁺ progenitors to orchestrate allergic rhinitis: A composite study
Source: PLoS One. 2026 Jun 22;21(6):e0351726. doi: 10.1371/journal.pone.0351726 (PMC13286145; doi:10.1371/journal.pone.0351726)
Supplement: S3 Fig — Western blot detection of CCR3 protein expression levels in mouse bone marrow cells. Note: *P < 0.05, **P < 0.01, ***P < 0.001, ****P < 0.0001, ns indicates P > 0.05, no statistical significance). (DOCX) [file pone.0351726.s014.docx]

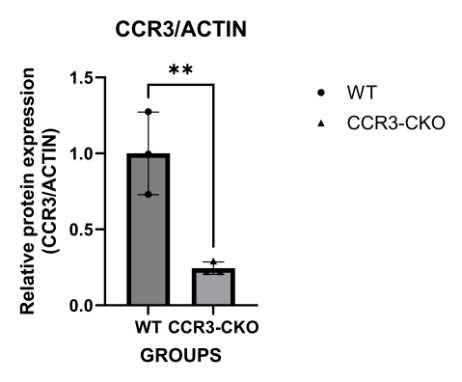

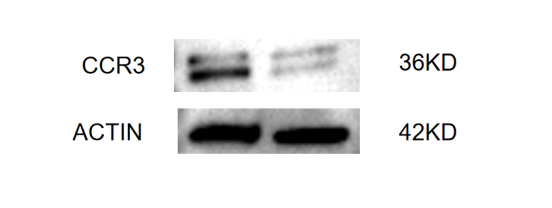


Supplementary Figure 3: Validation of knockout efficiency at RNA and protein levels

(a. qPCR detection of CCR3 mRNA expression levels in mouse bone marrow cells; b. Western blot detection of CCR3 protein expression levels in mouse bone marrow cells. Note: *P < 0.05, **P < 0.01, ***P < 0.001, ****P < 0.0001, ns indicates P > 0.05, no statistical significance)
